# Niagara University—Legal Opinion Regarding the Validity of Its Charter, by Sprague, Morey & Sprague, Attorneys, Buffalo

**Published:** 1884-05

**Authors:** 


### BUFFALO

### Medical and Surgical Journal.

VOL. XXIII. MAY, 1884. No. 10.

### Original Communications.

### NIAGARA UNIVERSITY.

THE LEGALITY OF THE CHARTER GRANTED BY THE REGENTS OF THE UNIVERSITY OF THE STATE OF NEW YORK, IN RELATION TO THE MEDICAL DEPARTMENT, AFFIRMED BY SPRAGUE, MOREY & SPRAGUE, ATTORNEYS, BUFFALO.

The question submitted is, has the Niagara University the power and authority to confer the degree of Doctor of Medicine upon the graduates from the Medical Department, which degree shall authorize such graduates to practice physic and surgery? By chapter 190 of the laws of 1863, Steven V. Ryan and others, and their successors, were constituted a body corporate by the name of the Seminary of Our Lady of Angels, the object of the said institution being declared by the first section of the act to be "to establish and maintain a seminary of learning in the county of Niagara, for the care and education of young men." Section 6 of this act reads as follows: "Whenever, in the opinion of the regents of the university, the state of literature of the said seminary and the value of its property (according to the regulations of the said regents) shall justify the same, the said regents may, on the petition of the trustees, by an instrument under their common seal, erect the said seminary into a college, with such name and such number of trustees, and on such conditions, and with such powers and privileges, conformable to law, as the said regents may deem proper." By an act passed March 12, 1883, entitled, "An act to amend chapter 190 of the laws of 1863, entitled 'An act to incorporate the Seminary of our Lady of Angels,'" the above-quoted 6th section was amended by adding at the end thereof the following words: "And the said college shall have the right to maintain any department of learning that is maintained by any college or university in this State, and may locate and maintain any department thereof in the county of Erie."

Pursuant to section 6, as so amended, the regents of the university on August 7, 1883, upon the petition of the trustees of the Seminary of Our Lady of Angels, issued their certificate or charter under their seal, erecting the said seminary into a college, as authorized by chapter 92 of the laws of 1883, under the name of Niagara University.

First. We are of the opinion that the provisions of section 6, amended as above by the act passed March 12, 1883, are broad enough to authorize the Niagara University to maintain a medical school or college, and to provide a faculty and to give medical instruction to students. There cannot be any doubt that the words "any department of learning," include in their meaning a department giving instruction in medicine; nor does there appear to be any doubt that on March 12, 1883, medical schools or departments were being maintained by colleges and universities in this State.

Second. We are of the opinion that the last clause in the amendment to section 6 authorizes the trustees of the Niagara University to locate and maintain such medical school as a department of said university in the county of Erie.

This would have been unlawful prior to the said act of 1883, under the provisions of section 21 of part 1st, chapter 14, title 7

of the Revised Statutes, which reads as follows: "Nor shall any college have or institute a medical faculty to teach the science of medicine in any other place than where the charter located the college."

The plain meaning and effect of the amendment of 1883, to section 6, is to except the college or university to be erected under that act from this provision. It cannot be construed to have any other meaning or application, and being the latest expression of the legislative will, it must control.

Third. The most important and difficult question yet remains to be answered. Has the Niagara University been authorized by law to grant and confer upon its graduates the degree of Doctor of Medicine, with the right to practice physic and surgery, by virtue of having received such degree?

By chapter 275 of the laws of 1844, all the legal prohibitions and penalties which had theretofore existed against the practice of physic and surgery by unlicensed persons were swept away, and no general enactment was thereafter adopted by the legislature upon that subject until May 9, 1880. By chapter 513 of the laws of 1880, it was provided as follows:

Section 1. "A person shall not practice physic and surgery within the State, unless he is twenty-one years of age, and either has been heretofore authorized so to do pursuant to the laws in force at the time of his authorization, or is hereafter authorized so to do, as prescribed by chapter 746 of the laws of 1872, or by subsequent section of this act."

Sec. 2 provides for the registration in the office of the County Clerk of every person then lawfully engaged in the practice of physic or surgery within the State, and of every person who should thereafter be duly authorized to practice physic and surgery.

Sec. 3 provides penalties for the violation of either of the two preceding sections.

Sec. 5 provides as follows: "The degree of Doctor of Medicine lawfully conferred by any incorporated medical college or university in this State, shall be a license to practice physic and surgery within the State after the person to whom it is granted shall have complied with section 2 of this act."

Chapter 740 of the laws of 1876, referred to in the first section of this act, provided for the appointment by the regents of the University of the State of New York of one or more boards of examiners in medicine, and required such examiners to faithfully examine the candidates referred to them for that purpose by the chancellor of said university, and furnish a detailed report in writing.

Section 6 of such act of 1872 reads as follows: "The regents of the university, on receiving the aforesaid reports of the examiners, and on finding that not less than five members of the board have voted in favor of a candidate, shall issue to him or her a diploma, conferring the degree of Doctor of Medicine of the University of the State of New York, which degree shall be a license to practice physic and surgery."

So that since the act of May 29, 1880, there are only two sources from which a person not then authorized to practice physic and surgery may obtain such authority: First, by submitting to an examination of a board of examiners appointed by the regents, and obtaining thereupon from the regents a diploma conferring the degree of Doctor of Medicine of the University of the State of New York. Or, second, by receiving the degree of Doctor of Medicine lawfully conferred by any incorporated medical college or university in this State.

Has the Niagara University the authority of law to confer such degree?

I. The provisions of section 6 of the act under which the university is incorporated, as amended by the act of 1883, do not expressly and in terms give this power. The words used are "and the said college shall have the right to maintain any department of learning that is maintained by any college or university in this State."

II. Section 6, of the act of 1883, provides that "the said regents may, on petition of the trustees, by an instrument under their common seal, erect the said seminary into a college with such name, and with such number of trustees, and on such conditions and with such powers and provisions conformable to law as the said regents may deem proper." The same power had been given to the regents in respect to academies generally by section 17 of chapter 59 of the laws of 1813, entitled "An act relative to the university." The words "with such powers and provisions conformable to law as the said regents may deem proper," obviously referred to the powers which had previously been conferred upon the regents in like cases. In the exercise of the power thus vested in them, the regents, in the charter granted by them to the Niagara University, declare that "in pursuance of the authority in us by law vested, do grant, ordain and declare that the said Seminary of Our Lady of Angels shall be, and is hereby erected into a college by the name hereinbefore mentioned for the instruction of youth in the learned languages, and in the liberal and useful arts and sciences." The charter then, after naming the incorporators, declares "that they and their successors shall be a body incorporate by the name of the Niagara University, and shall have, possess and enjoy all the powers and privileges, and be subject to such limitations and restrictions in all respects as are now or may be hereafter prescribed by the statutes of this State in regard to colleges, or by the ordinances or regulations of us, the said regents, in conformity to law."

It appears, therefore, that the Niagara University under this charter rightfully possesses all the powers and privileges which are possessed by any college in the State of New York.

The enquiry then is, have the colleges of this State the power and authority to grant the degree of Doctor of Medicine to their graduates? Chapter 59 of the laws of 1813, entitled "An act relative to the university," provides for the founding of colleges by the regents of the university; and in section 6, after providing for their incorporation, declares that such colleges shall have perpetual succession and enjoy all the corporate rights and privileges enjoyed by Columbia College in and by the act entitled, "An act to institute a university within this State," and for other purposes therein mentioned, passed April 13, 1787. On reference to this later act of 1787, it will be found that in section 10, in defining the power, authority, rights, privileges, franchises and immunities of Columbia College, provides that it shall be the same as those granted to the governors of the college of the Province of New York, in the State of New York, in America, by its charter of Oct. 31, 1754.

The powers granted by the charter of October 31, 1754, are in the following words: "And we do further of our especial grace, certain knowledge, and mere motion, will, give, and grant unto the said governors of the said college that, for the encouragement of the students of the said college to diligence and industry in their studies, they and their successors, and the major part of any fifteen or more of them convened and met together as aforesaid, do, by the president of the said college, give and grant any such degree and degrees to any of the students of the said college, or any other person or persons by them thought worthy thereof, as are usually granted by any or either of our universities or colleges in that part of our kingdom of Great Britain called England, and that the president, or such other persons to be appointed for that purpose as aforesaid, do sign and seal diplomas or certificates of such degree or degrees to be kept by the graduates as a testimonial thereof."

And by the provisions of section 6 of chapter 59 of the laws of 1813, these words above quoted define also the powers which were conferred upon Niagara University by the charter issued to them by the regents of the university.

The question, then, of the power of Niagara University to confer the degree of Doctor of Medicine, will be answered by ascertaining whether Columbia College enjoyed the right and privilege of conferring that degree under its charter of April 13,

1787. The power conferred by that charter by its reference to the charter of October 31, 1754, is in these words: "Give and grant any such degree and degrees to any of the students of said college, or any other person or persons by them thought worthy thereof, as are usually granted by any or either of our universities or colleges in that part of our kingdom of Great Britain called England."

It would probably be quite safe to rest upon the reasonable presumption that there was, in the year 1754, some university, or some college in England which granted the degree of Doctor of Medicine. The language quoted is, "Any or either of our universities or colleges;" so that if there was one such university or college in Great Britain having the power to grant the degree of Doctor of Medicine, then that power was conferred upon Columbia College by its charter of 1787. But we are not compelled to rest upon this presumption, though it appears to be a reasonable and safe one. The corporation named, the trustees of Columbia College was originally incorporated under the name of the governors of the colleges of the province of New York. The corporate name was changed to the trustees of Columbia College by section 8 of the law of April 13, 1787.

In a "historical sketch of Columbia College" in a book entitled "Statutes of Columbia College and its Associate Schools," printed for the college in 1880, this statement appears: "In August of the year 1767 a medical school was established." The same historical sketch quotes from an account of the institution supposed to be written by Dr. Cooper, its last president before the Revolutionary War, in the following words: "This seminary hath also produced a number of gentlemen who do honor to their professions, the place of their education and themselves, in divinity, law, medicine, etc., etc., in these and various other colonies." And the catalogue of Columbia College from 1754 to 1882, printed for the college in 1882, shows that from 1769 to 1810 various persons graduated from the medical department of Columbia College. This shows that it was the understanding of the trustees and faculty of the college department that under its charter of 1754, above quoted, it had the right to issue diplomas to persons graduating from its medical department.

III. The right and power to grant honors and degrees thus conferred upon all colleges chartered by the regents of the University is supplemented and strengthened by the provisions of the Revised Statutes; being article 2, of title 1, of chapter 15, of part 1st, entitled, "Of the powers and duties of the trustees of colleges."

Section 37 provides as follows: "Every diploma granted by such trustees shall entitle the possessor to all the immunities which by usage or statute are allowed to possessors of similar diplomas granted by any university, college, or seminary of learning in the United States."

It already appears that long before the adoption of the Revised Statutes, persons who had graduated from the medical department of Columbia College had attained distinction in the practice of medicine in New York and other colonies, and that "usage" had thus been established.

IV. This view of the proper construction of the statutes is also strongly confirmed by the fact that our statutes seem to have assumed that every lawfully incorporated college or university has the power to grant such honors and degrees. For example section 7 of chapter 59 of the laws of 1813, hereinbefore referred to, provides: "And be it further enacted that the said regents shall have the right of conferring by diplomas under their common seal on any person or persons whom they may think worthy thereof, all such degrees above or beyond those of Bachelor or Master of Arts, as are known to or granted by any university or college in Europe; and the degree of Doctor of Medicine granted by the said regents shall authorize the person on whom it is conferred to practice physic and surgery in this State, any law to the contrary notwithstanding."

This provision assumes as a fact that the degree of Doctor of Medicine is included in "such degrees as are known to and usually granted by any university or college in Europe." So in chapter 14 of part 1st of the Revised Statutes, section 21, title 7, provides as follows: "The degree of Doctor of Medicine conferred by any college in this State shall not be a license to practice physic or surgery." So this power to grant diplomas conferring the degree of Doctor of Medicine seems to have been assumed in chapter 45 of the laws of 1835, relating to Geneva College, and in chapter 25 of the laws of 1837, relating to the University of the City of New York. So, too, section 1st of chapter 436 of laws of 1874 provides that "every practitioner of medicine or surgery in this State, excepting licentiates or graduates of some medical society or chartered school, shall be required, and they are hereby commanded to obtain a certificate," etc.

Section 3 declares, "It is hereby declared a misdemeanor for any person to practice medicine or surgery in this State unless authorized so to do by license or diploma from some chartered school, State Board of Medical Examiners, or medical society, or who shall practice under cover of medical diploma illegally obtained." These sections seem to assume the right of a chartered school to grant a diploma which should afford evidence of the qualification of the person holding it to practice medicine. The same assumption runs through the provisions of chapter 513 of the laws of 1880, heretofore referred to.

V. The only reported case in this State which appears to have any bearing upon the question under consideration is that of The People vs. The Trustees of Geneva College, 5 Wendell reports, 211. The trustees of Geneva College—an incorporated college—located in Geneva, Ontario county, established a medical faculty in the City of New York, called the "Rutgers Medical Faculty of Geneva College," and claimed the right of issuing diplomas conferring the degree of Doctor of Medicine upon the graduates therefrom. This was an action in the nature of a *quo*

*warranto*, to test the power of Geneva College to exercise its corporate rights and franchises in this manner at the city of New York. The case was argued by Green C. Brownson and Jay for the people, and by Emmett and Ogden for the trustees. Chief Justice Savage, in delivering the opinion of the court, says: "The defendants are a corporation for the purpose of instructing pupils in the arts and sciences; they have power to confer degrees which are evidence of the proficiency made by their pupils; they have power, of course, to appoint tutors and professors to teach those branches of learning which will qualify pupils for receiving diplomas; these are franchises and cannot be exercised by a corporation without legislative authority; it is not denied that an individual may instruct pupils and may employ teachers under him, and may call them professors, and may also give a certificate or diploma; but a diploma or degree of Doctor of Medicine from an individual will not give its possessor a right to practice in this State as a physician; *a diploma or a degree from a college does give that right.* The individual who confers a degree does not profess to act by legislative authority; the corporation does. The degree of the individual confers no privilege or immunity upon the pupils; the degree of the corporation does confer a privilege; hence, the same act when done by an individual may be no franchise, yet is such when done by a corporation." To sum up we say, then:

I. That Niagara University is endowed by the law incorporating it, and by its charter, with all and any of the powers and privileges which belong to any legally incorporated college or university in the State of New York.

II. That the statute authorizing the regents of the university to incorporate colleges, and the Revised Statutes as well, confer upon the colleges of the State of New York the power to grant diplomas, which shall confer the degree of Doctor of Medicine.

III. The statute of the State from the time of the act incorporating the regents of the university down to 1880, have uniformly assumed that this power existed in the colleges of the State.

IV. In the only reported case bearing upon the question, Emmett, counsel, assumed, and the court decided that this power exists. There does not, therefore, appear to be any reasonable doubt that Niagara University has the power to grant diplomas conferring the degree of Doctor of Medicine; the right of a person who has received such a degree to practice physic and surgery is given by chapter 513 of the laws of 1880.

Dated Oct. 1, 1883.

